# Innate Immunity in the Persistent Inflammation, Immunosuppression, and Catabolism Syndrome and Its Implications for Therapy

**DOI:** 10.3389/fimmu.2018.00595

**Published:** 2018-04-04

**Authors:** Hiroyuki Horiguchi, Tyler J. Loftus, Russell B. Hawkins, Steven L. Raymond, Julie A. Stortz, McKenzie K. Hollen, Brett P. Weiss, Elizabeth S. Miller, Azra Bihorac, Shawn D. Larson, Alicia M. Mohr, Scott C. Brakenridge, Hironori Tsujimoto, Hideki Ueno, Frederick A. Moore, Lyle L. Moldawer, Philip A. Efron, The Sepsis and Critical Illness Research Center Investigators

**Affiliations:** ^1^Department of Surgery, University of Florida College of Medicine, Gainesville, FL, United States; ^2^Department of Surgery, National Defense Medical College, Tokorozawa, Japan; ^3^Department of Medicine, University of Florida College of Medicine, Gainesville, FL, United States; ^4^The Sepsis and Critical Illness Research Center, University of Florida College of Medicine, Gainesville, FL, United States

**Keywords:** sepsis, chronic critical illness, persistent inflammation/immunosuppression and catabolism syndrome, innate immunity, inflammation

## Abstract

Clinical and technological advances promoting early hemorrhage control and physiologic resuscitation as well as early diagnosis and optimal treatment of sepsis have significantly decreased in-hospital mortality for many critically ill patient populations. However, a substantial proportion of severe trauma and sepsis survivors will develop protracted organ dysfunction termed chronic critical illness (CCI), defined as ≥14 days requiring intensive care unit (ICU) resources with ongoing organ dysfunction. A subset of CCI patients will develop the persistent inflammation, immunosuppression, and catabolism syndrome (PICS), and these individuals are predisposed to a poor quality of life and indolent death. We propose that CCI and PICS after trauma or sepsis are the result of an inappropriate bone marrow response characterized by the generation of dysfunctional myeloid populations at the expense of lympho- and erythropoiesis. This review describes similarities among CCI/PICS phenotypes in sepsis, cancer, and aging and reviews the role of aberrant myelopoiesis in the pathophysiology of CCI and PICS. In addition, we characterize pathogen recognition, the interface between innate and adaptive immune systems, and therapeutic approaches including immune modulators, gut microbiota support, and nutritional and exercise therapy. Finally, we discuss the future of diagnostic and prognostic approaches guided by machine and deep-learning models trained and validated on big data to identify patients for whom these approaches will yield the greatest benefits. A deeper understanding of the pathophysiology of CCI and PICS and continued investigation into novel therapies harbor the potential to improve the current dismal long-term outcomes for critically ill post-injury and post-infection patients.

## Introduction

The phenotype of the critically ill patient is evolving. While historically a significant number of these patients would have succumbed to early death, recent diagnostic and therapeutic advances allow many of these patients to survive the acute phase of their disease (1, 2). In addition, the modern physician can compensate for the organ failure suffered by many intensive care unit (ICU) patients after the acute phase of an insult, resulting in a decreased hospital mortality (1–3). A prime example of this phenomenon exists among severely injured trauma patients who require prolonged utilization of ICU resources. Advances in early hemorrhage control and physiologic resuscitation strategies for critically ill trauma patients have shifted the frequency distribution of post-injury mortality away from early in-hospital death toward late death (4–6). Septic patients have experienced a similar phenomenon. Sepsis is a complex physiologic condition that represents a major public health challenge. Sepsis is the most expensive hospital condition treated in the United States, with costs exceeding $27 billion annually (REF; https://hcupnet.ahrq.gov/#setup). Over the past 30 years, attempts to treat the “cytokine storm” of sepsis have universally failed to improve outcomes. In addition, no single biological response modifier has been approved by the FDA for treating sepsis. However, the “Surviving Sepsis Campaign” initiated in 2002 promotes earlier recognition of sepsis and implementation of best practices that, as now recognized, results in lower in-hospital mortality for patients with sepsis (7). Unfortunately, similar to trauma, improved early survival has led to a new patient phenotype described as chronic critical illness (CCI) (8). The definition of CCI includes ICU length of stay (LOS) greater than or equal to 14 days with evidence of persistent organ dysfunction, measured using components of the Sequential Organ Failure Assessment (SOFA) score at 14 days. According to this definition, approximately 40% of sepsis patients progress to CCI (9). These sepsis survivors are commonly discharged from the hospital to long-term acute care facilities (LTACs) and skilled nursing facilities where they often fail to rehabilitate and succumb to indolent death (1, 8). We believe that individuals who experience a morbid post-ICU hospital course are developing a new syndrome, termed the persistent inflammation, immunosuppression, and catabolism syndrome (PICS) (1). Emerging evidence indicates that the pathogenesis of PICS involves chronic low-grade inflammation, suppressed host protective immunity, and loss of lean tissue (10–13). This review focuses on the role of emergency myelopoiesis and innate immunity in trauma and sepsis and the ontogeny of critically ill patients with CCI and PICS.

## Pathogen Recognition and the Innate Immune Response

### Pathogen Recognition

**Figure 1:**
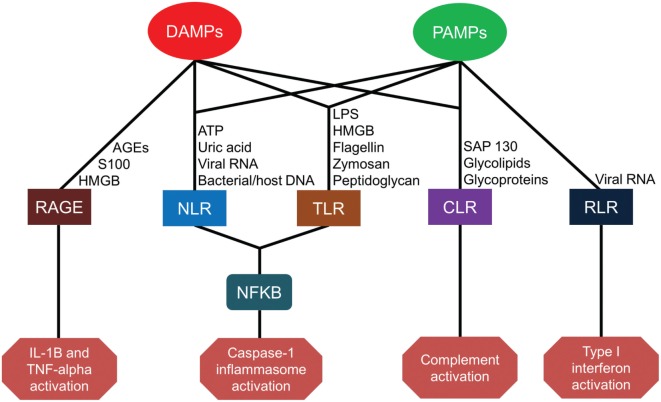
Pattern-recognition receptor pathways for damage-associated molecular patterns (DAMPs) and pathogen-associated molecular patterns (PAMPs). AGEs, advanced glycosylation end products; HMGB, high-mobility group box; ATP, adenosine triphosphate; RNA, ribonucleic acid; DNA, deoxyribonucleic acid; LPS, lipopolysaccharide; RAGE, receptor for advanced glycation end products; NLR, nucleotide-binding oligomerization domain-like receptors; TLR, toll-like receptors; CLR, C-type lectin receptors; RLR, retinoic-acid-inducible gene-I-like receptors; NF-kB, nuclear factor kappa-light-chain-enhancer of activated B cells; IL, interleukin; TNF, tumor necrosis factor.

Severe injury or infection leading to CCI and PICS begins with the recognition of alarmins, primarily consisting of microbial products and damaged tissue (14). The innate immune system relies on germ line-encoded pattern-recognition receptors (PRRs) to sense components of foreign pathogens and damaged cells to mount host-protective responses (15). These PRRs are expressed on a variety of host cells, including cells of myeloid, endothelial, and epithelial lineages. These PRRs detect conserved microbial components called pathogen-associated molecular patterns (PAMPs) as well as host molecules derived from damaged cells, known as damage-associated molecular patterns (DAMPs). Major classes of PRRs include Toll-like receptors (TLRs), C-type lectin receptors (CLRs), nucleotide-binding oligomerization domain (NOD)-like receptors (NLRs), retinoic-acid-inducible gene-I (RIG-I)-like receptors (RLRs), and receptor for advanced glycation end products (RAGE) (15). The sheer number, diversity, and redundancy of these pathogen-recognition receptors emphasize their essential role in host-protective immunity (Figure 1).

Toll-like receptors (TLRs) are the most broadly studied PRRs. To date, 13 different TLRs have been identified in humans and mice, with slight differences in receptor type and function between species. TLR1–TLR9 are functional in both species, but TLR10–13 is not conserved between species. While some TLRs are expressed on the plasma membrane (TLRs 1, 2, 4, 5, and 6) to constantly sample the local environment, others are located within endosomal compartments (TLRs 3, 7, 8, 9, 11, 12, and 13) to sense host danger signals or microbial proteins and nucleic acids (15). TLRs located on the plasma membrane detect external microbial components and circulating damage signals such as lipopolysaccharide (LPS), phospholipids, zymosan, flagellin, peptidoglycan, S100A8/9, and endogenous high-mobility group box (HMGB) nuclear proteins from distressed cells (16–22). TLRs located in cytoplasm, however, detect viral or microbial nucleic acids as well as the mitochondrial nucleic acids associated with cell injury (23). TLRs play a central role in initiating the innate immune response in cooperation with other PRRs through diverse and overlapping signaling pathways (24).

Pathogen recognition also occurs through C-type lectin receptors (CLRs). CLRs are carbohydrate-binding transmembrane proteins expressed by antigen-presenting cells (APCs). CLRs are able to recognize glycolipids and glycoproteins present on numerous pathogens including bacteria, parasites, fungi, and viruses, as well as on host cells (25). Furthermore, the CLR macrophage-inducible C-type lectin receptor (Mincle) can detect the DAMP spliceosome-associated protein 130 (SAP-130) released by necrotic cells (26).

Nucleotide-binding oligomerization domain-like receptors are a family of more than 20 cytoplasmic receptors. NOD1 and NOD2 were the first identified NLRs capable of recognizing bacterial peptidoglycan moieties and triggering inflammation by activating nuclear factor (NF)-kB and mitogen-activated protein kinase pathways. Recent studies have demonstrated that NOD2 is also capable of sensing viral ribonucleic acid (RNA) (27, 28). Other receptors, such as the NLR family pyrin domain containing 3 (NLRP3), detect not only bacterial peptidoglycan but also bacterial flagellin, cytosolic microbial and host deoxyribonucleic acid (DNA), as well as DAMPs, such as adenosine triphosphate and uric acid. These pathogen and damage signals lead to the formation of large protein complexes in the cytosol, termed inflammasomes, which subsequently active caspase-1. Activated caspase-1 in the inflammasome converts the pro-inflammatory cytokines (ILC) interleukin (IL)-1β and IL-18 into their biologically active forms. In addition, caspase-1 activity induces inflammatory cell death in infected myeloid cells in a process known as pyroptosis (29).

Retinoic acid-inducible gene-I-like receptors are cytoplasmic receptors that comprise RIG-I, melanoma differentiation-associated protein 5 (MDA5), and laboratory of genetics and physiology 2 (LGP2). RIG-I and MDA5 recognize viral double-stranded (ds) RNA and activate the innate immune response (30), whereas LGP2 is reported to play both negative and positive regulatory roles in RIG-I and MDA5 signaling (31, 32). RIG-I and MDA5 bind to short dsRNA (<1 kbp) and longer dsRNA (>1 kbp), respectively.

Finally, RAGE is a transmembrane receptor expressed on human endothelial cells, monocytes, and lymphocytes. RAGE binds to several ligands including advanced glycation end products (AGEs) from aging erythrocytes as well as HMGB1 and S100 proteins. Interactions between AGEs and RAGE on endothelial cells induce oxidative stress and mitochondrial dysfunction leading to tissue injury (33). HMGB1–RAGE interactions also contribute to cell proliferation, cell migration, and angiogenesis (34). Engagement of S100 protein with RAGE is associated with an increased expression of the pro-ILC IL-1β and tumor necrosis factor-alpha (TNF-α) (35).

### Signaling Pathway

Upon host recognition of PAMPs or DAMPs, PRRs initiate a complex set of downstream signaling events that induce a host-protective response. This includes recruitment and phosphorylation of intracellular intermediates leading in part to the activation of immediate response genes. PRR-signaling pathways are wide ranging and often redundant. For example, the TLR signal transduction employs a Toll/IL-1 receptor domain, which has five adaptor proteins: myeloid differentiation primary response 88 (MyD88), Toll-IL-1 receptor domain containing adaptor protein (TIRAP), Toll-IL-1 receptor domain containing adaptor protein inducing interferon (IFN) β (TRIF), TRIF-related adaptor molecule (TRAM), and sterile-α and armadillo motif containing protein (SARM) (36). Most TLRs (TLR-1, TLR-2, and TLR-4–9) utilize the MyD88-dependent pathway, whereas TLR-3 and TLR-4 utilize the TRIF-dependent pathway. The TRIF pathway activated by TLR-3 or TLR-4 tends to stimulate the expression of type-I IFNs rather than ILCs. Regardless of the proximal adaptor proteins utilized, TLR signaling terminates in the activation of the transcription factors nuclear factor-kappa B (NF-κB), IFN regulator factor 3/7, and activator protein-1 (AP-1). In addition, TLR signaling induces the secretion of pro-ILCs, type-I IFN, chemokines, and antimicrobial peptides (37). Furthermore, different PRRs may sense the same PAMP. Microbial pathogens are generally composed of several PAMPs, which bind to multiple PRRs simultaneously. Therefore, each PRR can potentiate the recognition of “non-self” antigens to mount an effective immune response against infection.

### Innate Immune Response

#### Cellular Response

**Figure 2:**
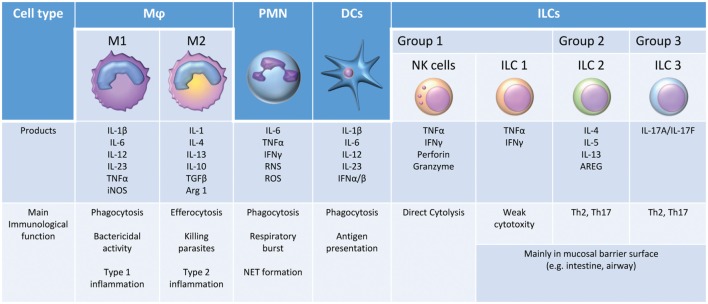
The main immunological functions and products of innate immune cells.

Pattern-recognition receptors activation and downstream signaling result in both nonspecific and pathogen-specific host cellular responses to prevent or eliminate host stressors, such as microbial infection or tissue damage (38). The suppression of microbial replication, tissue invasion, and dissemination from the site of infection involves multiple innate immune cells: neutrophils (PMNs), monocytes/macrophages (Mφ), dendritic cells (DCs), natural killer (NK) cells, and innate lymphoid cells (ILCs) (Figure 2). All these cells can play a crucial role in the early inflammatory response (39). PMNs primed by cytokines such as TNF-α eliminate invading pathogens through phagocytosis, the production of reactive oxygen species (ROS), and the release of neutrophil extracellular traps (NETs) in a process known as NETosis (40). Mφ also contribute significantly to the host defense against infection. M1 Mφ, or classically activated Mφ, are activated by microbial stimuli alone or in combination with other endogenous or exogenous inflammatory signals, such as IFN-γ and LPS, respectively, to produce high levels of pro-ILCs (e.g., IL-6, IL-12, and TNF-α), ROS, and reactive nitrogen species. These early cytokine responses serve two primary purposes: (1) to signal the host regarding the type and magnitude of the infection and (2) to delay microbial expansion and colonization until T- and B-lymphocytes are able to initiate an adaptive immune response, which ultimately contributes to pathogen elimination. DCs are known as professional APCs (41). DCs present pathogen-derived antigenic peptides to CD4^+^ T cells in lymph nodes, leading to the activation and differentiation of antigen-reactive T-effector cells (42). The NK cell is also an effector cell of innate immunity, producing cytokines such as IFN-γ during the early phase of inflammation. In addition, these cells have a direct cytotoxic activity *via* the induction of apoptosis from the release of perforin, granzymes, TNF-α, Fas ligand (FasL), and TNF-related apoptosis-inducing ligand (TRAIL) (43). The crucial role of NK cells in sepsis and trauma is increasingly being recognized (44). Finally, ILCs are another newly described population of innate immune cells. ILC1 cells are weakly cytotoxic but can express several ILCs. ILC2 and ILC3 cells are thought to promote T_H_2 and T_H_17 responses, respectively, and are critical for the crosstalk between innate and adaptive immunity (45).

#### Coagulation System

The innate immune system also incorporates cells and systems beyond effector white blood cells. In a further attempt to control local infections or tissue damage, an endothelial cell-target hypercoagulable state occurs with the presumed intent of reducing blood loss and trapping microbial pathogens (46). This response occurs *via* multiple pathways (47). Tissue factor (TF), an important trigger of coagulation, is upregulated and decrypted in response to chemical or physical damage, cytokines (such as TNF-α and IL-1β), and infectious agents (48). TF upregulation leads to the activation of platelets and induction of protease-activated receptor-mediated signaling, resulting in the production of additional cytokines and the expression of cell adhesion molecules. These responses result in the formation of NETs within the vasculature, which bind platelets and promote thrombosis (49). Moreover, platelet activation within the sites of bacterial infection also provides a first line of defense against pathogenic microbial agents (50). Although activated platelets can have bactericidal activity through platelet secretion, platelets contribute more to bactericidal activity by forming platelet-bound bacteria bundles, which boost the activity of professional phagocytes such as PMNs and Mφs (51, 52). Furthermore, recent studies suggest that TLRs and the complement system contribute to coagulation and thrombosis (53, 54). Platelet-derived TLR-4 has been shown to upregulate TF expression in LPS-induced endotoxemia and microvascular thrombosis in mice (54). Extracellular histones that are released from dying cells contribute to platelet activation and increase coagulation *via* TLR-2 and TLR-4 signaling (55).

#### Complement System

The complement system is a major component of the innate immune system that has the ability to discriminate self from non-self (or damaged/altered self) and eliminate pathogens that might harm the host. The complement system is a complex network of more than 50 proteins in the plasma and on cell surfaces arranged in a system of proteolytic cascades (56). There are three major complement pathways: the classical pathway, the lectin pathway, and the alternative pathway (57). The classical and lectin pathways are activated with the help of PRRs, whereas the alternative pathway is activated spontaneously without the need for PRRs (58). Regardless of pathway, the complement system culminates in three broad effector pathways: (1) the generation of potent pro-inflammatory anaphylatoxins, (2) opsonization with targeting of pathogen surfaces with complement opsonins, and (3) the direct lysis of targeted pathogens by the formation of membrane-penetrating pores, termed the membrane attack complex (MAC) (57).

Anaphylatoxins (C3a, C4a, and C5a) are the activation products of the effector phase of the complement system. Anaphylatoxins are traditionally reported to have diverse pro-inflammatory effects. This includes smooth muscle contraction, an increased leukocyte chemotaxis, vasodilation, an increased vascular permeability, an improved neutrophil oxidative burst, an enhanced phagocytosis, and an increased release of inflammatory mediators like histamine (57). In addition, recent studies have demonstrated that anaphylatoxins are potent mediators of numerous other biological responses, such as the modulation of cytokine expression, increasing the expression of adhesion molecules on neutrophils, the activation of the coagulant pathway, and the regulation of adaptive immune cells (59–64).

Complement-mediated opsonization plays a major role in phagocyte pathogen recognition. Complement component C3 is cleaved into C3a and C3b by enzymes in all three pathways of complement activation. This cascade results in a conformational change of C3b that allows the covalent association of C3b with the pathogen surface and subsequent cleavage by factor I and its co-factor. Moreover, this conformational rearrangement exposes numerous binding sites for receptors. There are complement C3 receptors including CR1–4 and CRIg that recognize C3b and its fragments. These receptors trigger phagocytosis, promote erythrocyte transportation and clearance, and enhance B cell immunity and regulate T-cell proliferation (65–67).

The MAC is the pore-forming terminal assembly of the complement system (C5b–C9), which leads to cell lysis on the surface of bacteria and other targets. The MAC has diverse roles, including the modulation of cell proliferation and the activation of the inflammatory response. A recent study reported that the MAC on a Rab5^+^ endosome could activate noncanonical pathways for NF-κB activation through intracellular signaling and promote the inflammatory response (68).

#### Bone Marrow (BM) and Myelopoiesis

Although the early host response is recognized immediately at the site of infection or tissue injury by resident innate cell populations, the recruitment of additional innate immune cells is almost immediate. Large numbers of mature myeloid cells are released from the BM and are recruited to the site of infection or injury (69, 70). Although many chemokines are involved in the recruitment of innate immune cells, one of the most important is C-X-C motif ligand 12 (CXCL12), also known as stromal cell-derived factor 1 (SDF-1) (71). Under normal conditions, high levels of CXCL12 in the BM play a critical role in innate immune cell chemotaxis and retention. However, in response to a host challenge, BM CXCL12 concentrations rapidly decline, and increased levels are seen at the site of infection or inflammation (72, 73). This reverse gradient appears to be influenced by the neuroendocrine stress response and may serve as a primary signal for the massive efflux of cells from the BM. In addition, multiple chemokines are required for this integrated recruitment of specific leukocytes at certain times post sepsis or trauma (69, 70). In the immediate period, BM niches are created by the exiting cells, and these niches are sensed by mesenchymal cells that reside in the bone and BM. Subsequently, there is a rapid release of proteins, such as FMS-like tyrosine kinase-3 ligand (Flt3L), which drives the proliferation of hematopoietic stem cells (HSCs), primarily of the short-term (ST-HSCs) subtype (74, 75). Thus, the numbers of BM lineage-negative, Sca-1 positive, c-kit-positive cells, multipotent progenitor cell (MPP) 1s and MPP2s greatly expand in the acute period following the insult (13).

Interestingly, developing MPPs do not differentiate equally along myeloid, lymphoid, and erythroid populations. Rather, the cytokine milieu present during infection or tissue damage drives myelopoiesis at the expense of both erythropoiesis and lymphopoiesis (76–78). While colony-stimulating factor 1, IL-3, and granulocyte colony-stimulating factor (G-CSF) concentrations rise dramatically, the compensatory erythropoietin response is blunted and IL-7 production is markedly suppressed. The end result is a preferential differentiation toward myelopoiesis. Consequently, BM is composed of nearly 95% myeloid cells within several days of sepsis (79). The resulting lymphopenia is exacerbated by T-cell dysfunction and increased lymphocyte apoptosis, and the resulting anemia is exacerbated by hepcidin-mediated iron restriction that is minimally responsive to exogenous iron and erythropoietin administration (79–81). These events have a pathophysiologic synergy.

Severe anemia among critically ill patients is often managed with allogeneic red blood cell transfusion. This can lead to transfusion-related immunomodulation and increased susceptibility to nosocomial infection in a population already with dysfunctional innate and adaptive immune systems (82, 83). Therefore, the rapid expansion of immature myeloid cells produced in lieu of lymphocyte and erythrocyte progenitors is being studied since its modification could potentially correct several pathologies in the CCI population (10, 11).

Many of the myeloid cells in the BM never reach a fully differentiated state. They are released in large numbers as immature myeloid cells. Although they are phenotypically differentiated to granulocyte-like and monocyte-like cells, their cellular function is dissimilar to that of terminally differentiated innate immune effector cells (84, 85). The pathophysiology of failure of immature myeloid cells to differentiate into mature granulocytes, monocytes, and DCs is complex, with contributions by the cytokine and endocrine milieu associated with microbial recognition or tissue damage. Gabrilovich et al. demonstrated that immature myeloid splenocytes from tumor-bearing animals with active inflammation differentiated into DCs and macrophages after being injected into healthy mice while these injected cells remained immature after being placed into other tumor-bearing mice (86).

The functions of these immature myeloid cells and their role in the sepsis and trauma response are poorly understood (87). Clearly, one fraction of these cells is the myeloid-derived suppressor cell (MDSC) population that has been initially described in the cancer population (88). Currently, two types of MDSCs have been identified, granulocytic and monocytic, both representing their phenotype and their ontology. Regardless, both these cells suppress T-effector cells and weaken the effectiveness of immunotherapies targeting T-cell activation (89, 90). The MDSC mechanisms of T-effector and antigen-presentation cell suppression are multifold and differ between the granulocytic and monocytic subtypes. These mechanisms include, but are not limited to, the production of the immune-suppressive cytokines, TGF-β and IL-10, the nitrosylation of key proteins on the T-cell receptor, arginine depletion, and the increased expression of T-cell checkpoint inhibitors, including programmed cell death protein ligand 1 (PD-L1), cytotoxic T-lymphocyte associated protein 4 (CTLA4), and B- and T-lymphocyte attenuator (88, 91–93).

MDSCs have a strong phagocytic activity, but are poor antigen presenters, and produce increased amounts of pro-ILCs (e.g., TNF-α and MIP-1α/CCL3), superoxides, and nitric oxide (79, 94). Cuenca et al. demonstrated that the early expansion of MDSC in mice bearing small tumors protected mice from infectious challenges, but massive expansion associated with larger tumor burdens made the animals exquisitely sensitive to both microbial infection and exogenous PAMPs (95).

Among critically ill septic patients, the proportion of circulating MDSCs correlates with the magnitude of the inflammatory response and predicts hospital trajectory and long-term clinical outcomes (10, 11). Septic patients with persistent MDSC expansion have a longer ICU LOS, a greater in-hospital mortality, and are more likely to be discharged to a rehabilitation facility compared with patients for whom MDSC populations return to baseline within 2 weeks (10). Therefore, it appears that MDSC persistence, rather than the initial MDSC expansion, may contribute to the pathophysiology of CCI and PICS.

#### Phagocytosis and Antigen Presentation

Phagocytosis is a central process in innate immunity that eliminates pathogenic microbial agents and facilitates the presentation of antigens to adaptive immune cells (96). Moreover, this process also contributes to the maintenance of normal cellular homeostasis and wound healing of the host organism (97–99). Phagocytes can be classified into two groups, specifically professional and non-professional phagocytes (100). Professional phagocytes (i.e., neutrophils, monocytes, macrophage, DCs, osteoclasts, and eosinophils) play a major role in pathogen elimination and antigen presentation (96, 101, 102). Non-professional phagocytes, including epithelial cells and fibroblasts, use this process to help maintain homeostasis (103, 104).

To initiate the process of phagocytosis, a direct physical contact between the phagocyte and the target must occur. Residual phagocytes extend membrane protrusions to detect and capture the target (105). Circulating phagocytes then recognize released attractant signals, mostly chemokines, from the site of infection and then migrate to that area (106). Subsequently, various receptors on the phagocytes bind to their ligands on the target and activate the signaling cascade of phagocytosis in the host cell. These receptors are classified as non-opsonic receptors and opsonic receptors. Non-opsonic receptors, such as C-type lectins (Dectin-1, Dectin-2, and Mincle) and scavenger receptor A, can recognize PAMPs as well as ligands on apoptotic cells, whereas opsonic receptors, such as FcR, CR1–4, and CRIg, can recognize the opsonized particles (107). The process of phagocytosis results in the elimination of internalized targets by maturation of the phagosome and fusion of the endosome and lysosome (108). In addition to phagolysosome activity, NADPH-oxidase assembly occurs within the phagosome, and various products such as reactive oxygen intermediates, elastase, and cytokines are synthesized and released into the phagosome where the microbes are killed and the microbial products/antigens are processed (108).

Phagocytes also play an important role in antigen presentation, and as mentioned previously, DCs are especially relevant to this process (41). DC phagosomes engulf the pathogens and perform incomplete proteolysis. The resulting products, which are a suitable size for binding with the major histocompatibility complex molecule (MHC), are presented to lymphoid cells at the plasma membrane (109). Engulfed antigens are then loaded into and associated with MHC II or MHC I molecules and displayed on the cell surface for the activation of lymphoid cells in a process termed cross-presentation (110). In the maturation process, phagocytosis triggers downregulation of their phagocytic capacity (111), and mature DCs migrate to lymph nodes and work primarily as APCs (112). There is a specific loss and alteration in the function of DCs after sepsis, and this contributes to subsequent host immune dysfunction (113–115).

Regarding toxin and pathogen clearance, the liver is one of the most important organs of host defense. In particular, Kupffer cells (KCs) play a major role in immune surveillance, including pathogen identification as well as antigen presentation (116). KCs represent 80–90% of all tissue macrophages in the entire body and are located in the sinusoid of the liver, adherent to liver sinusoidal endothelial cells (117). KCs phagocytose pathogenic microbial agents through TLRs, complement receptors, and antibody receptors (116). After ingesting pathogenic microbial agents, KCs produce several pro-ILCs and chemokines, which attract NKT cells, neutrophils, and T cells to present microbial antigens, as well as activate these cells for eliminating pathogenic microbial agents (118–120). KCs also phagocytose host cells such as neutrophils and platelets, subsequently producing either pro-ILCs or anti-ILCs to resolve inflammation (117, 121–123).

#### Apoptosis, Necrosis, and Pyroptosis

Apoptosis is a non-lytic and usually immunologically silent form of cell death characterized by cell shrinking, chromatin condensation, and membrane blebbing (124). Apoptosis is known to play a major role in physiologic development and tissue homeostasis (125). This process occurs through two mechanisms, known as the extrinsic and intrinsic pathways (126). The extrinsic pathway is mediated by death receptors such as TNF receptor 1, Fas, and TNF-related apoptosis-inducing ligand receptors that lead to the subsequent activation of caspase-8 activation. The intrinsic “mitochondrial” pathway, however, activates caspase-9. Regardless, both pathways culminate in the activation of caspase-3 (127–130). Moreover, apoptosis can also occur through granzymes released by cytotoxic T lymphocytes and NK cells (131). Although apoptotic cell death itself is immunologically silent, apoptotic cells can be engulfed by Mφs and DCs which induces the release of anti-ILCs such as IL-10 and TGF-β as well as reducing the release of TLR-4 mediated pro-ILCs. Both of these effects can promote the resolution of inflammation (123, 132, 133). Furthermore, the uptake of apoptotic DCs induces the differentiation of forkhead box P3 (FoxP3)^+^ regulatory T cells (134). In sepsis, apoptotic cell death is increased in multiple cell types except for PMNs. This includes apoptotic-induced depletion of splenic follicular DCs, B cells, and CD4^+^ T cells in septic patients (80, 135–137). These anti-inflammatory effects of apoptosis are thought to contribute to early and late poor clinical outcomes after sepsis (138, 139).

In contrast to apoptosis, necroptosis is a lytic cell death and thought to result in the release of DAMPs, which leads to immune system activation and extensive inflammation like pyroptosis (140). Necroptosis is initiated by death receptors, such as TNF receptor 1, Fas, and TNF-related apoptosis-inducing ligand receptors, and PRRs including TLR-3, TLR-4, and Z-DNA sensor DAI (141). The downstream activation of these receptors usually results in NF-κB activation and/or apoptosis. However, the necroptotic pathway can also be induced when caspase-8 activity is actively inhibited by viral-derived caspase inhibitors. Therefore, the function of necroptosis is thought to be as a backup cell death program that is induced when apoptosis induction fails (142).

Pyroptosis is a lytic form of programmed cell death, which is activated by both the canonical and noncanonical inflammasome. Pyroptosis can be initiated in response to the recognition of PAMPs and DAMPs through PRRs such as NLRs and AIM2 in cytosol. These PRRs form inflammasomes and activate inflammatory caspases that induce pro-ILCs (e.g., IL-1β and IL-18) and pyroptosis. Pyroptosis enhances the inflammatory response through the release of pro-ILCs and improved microbe capture in the phagocyte (143, 144). Pyroptosis also contributes to cell death and cessation of other processes such as phagocytosis and antigen presentation. Thus, the balance between the initiation of pyroptosis and the promotion of inflammation while retaining viability is important for host immunity.

Pyroptosis has features that are distinct from necrosis and apoptosis and have important implications for the innate immune response to trauma and sepsis. Apoptosis describes a highly regulated cell death pathway involving multiple caspases in the relative absence of inflammation, whereas necrosis involves the disintegrations of cell membranes with spillage of DAMPs, cell contents, and cell membrane components into the interstitial space, promoting the inflammatory response (145, 146). Necrosis may occur in a more regulated fashion termed necroptosis, resulting in a more controlled local inflammatory response (147). By contrast, pyroptosis is a tightly regulated form of cell death that is also associated with a significant inflammatory response (148). Pyroptosis may offer adaptive advantages for patients with sepsis by promoting bacterial clearance *via* pore-induced intracellular traps (144).

## Sepsis is Characterized by Dysregulation and Overactivation of Host-Protective Innate Immunity

Host immune responses to pathogens depend on the magnitude of the physiologic insult. Highly virulent pathogens and host immune impairment may potentiate the host’s vulnerability to sepsis. Sepsis is defined as a life-threatening organ dysfunction caused by a dysregulated host response to an infection (149). An overactivated or a dysregulated innate immune response to infection can result in tissue damage, cellular compromise, and molecular dysregulation that lead to organ dysfunction and failure.

### Coagulation in Sepsis

Although the activation of the coagulation cascade is protective in reducing the dissemination of invading pathogens through fibrin deposition (150, 151), overactivation of coagulation and subsequent microthrombus formation may lead to disseminated intravascular coagulation (DIC) which can reduce microcirculation and oxygen delivery to tissues (152). This can result in multiple organ failure (153). The prevalence of DIC in severe sepsis has been reported to be as high as 47% (154). During sepsis-induced activation of coagulation, the function of anticoagulant pathways such as antithrombin, activated protein C, and TF pathway inhibitor (TFPI) can be impaired (155). These impairments lead to increased fibrin formation and insufficient fibrinolysis, which results in microvascular thrombus.

### Complement Activation in Sepsis

The complement system is activated during sepsis, resulting in increased levels of C3a, C4a, and C5a in plasma (156, 157). Excessive amounts of these anaphylatoxins can lead to adverse systemic consequences through several mechanisms, including, but not limited to, hemodynamic instability and impaired oxygen delivery (158). In the complement system, C5a is the most powerful inflammatory mediator, and the C5a–C5aR interaction can induce various biological responses. In acute sepsis, C5a plays a central role in the production of both inflammatory and anti-ILCs (159). In addition, the excessive generation of C5a results in the aggravation of the cardiovascular system as well as paralysis of crucial neutrophil functions, such as chemotaxis, respiratory burst, and phagocytosis (160, 161). Moreover, C5a regulates the host reaction to sepsis (61, 162, 163).

### Neutrophil Priming and NETs in Sepsis

PMNs exist in three states: resting, primed and activated. PMNs shift from a resting state in the circulation to an activated state at the site of infection *via* several priming stimuli, including, but not limited to, cytokines, LPS, and C5a (164–166). During sepsis, excessive priming of PMNs can cause excessive production of ROS, which are released into the nearby environment, resulting in tissue damage (167). Priming of the neutrophil respiratory burst is believed to be involved in the pathophysiology of the acute respiratory distress syndrome. In addition, NETs can harm the host during sepsis. Although NET formation can help eradicate a wide range of pathogens in the early phase of the infection, the generation of excessive amounts of NETs, as well as impairment of their degradation by extracellular DNAases within the bloodstream, can damage tissues and endothelia (168). This can induce diffuse thrombosis and lead to DIC and acute organ injury (169, 170). Also, extracellular histones released by excessively accumulated NETs are cytotoxic to endothelial and epithelial cells (171, 172).

### MDSCs in Sepsis

As mentioned above, MDSCs are a heterogeneous population of immature myeloid cells with the common ability to induce immunosuppression. MDSCs are found in healthy individuals at a low amount in peripheral blood (10), but expand dramatically in cancer, autoimmune diseases, inflammation, and sepsis (10, 79, 173–175).

Myeloid-derived suppressor cell expansion is thought to be primarily mediated by the Janus kinase protein family leading to the activation of transcription 3 (STAT3). Activation is a second step dependent primarily on NF-κB activation through the MyD88 pathway. Several inflammatory mediators, such as IL-6, G-CSF, GM-CSF, and VEGF, are involved in the latter pathway (79, 176–179). Moreover, activated MDSCs are upregulated by S100A8/9. Following activation, MDSCs produce multiple mediators such as ROS, inducible nitric oxide synthase, arginase-1, TGF-β, and IL-10 that suppress T-effector and NK cell proliferation and activation, preferentially induce Th2 polarization (180–182). Activated MDSCs from septic mice have been demonstrated to produce several pro-inflammatory factors such as TNF-α, RANTES, and MIP-1β in response to LPS administration (79).

Unlike the well-defined role of MDSCs in cancer, the role of MDSCs in sepsis is still controversial. MDSC expansion and immunosuppressive functions are observed in both septic mice and humans (10, 11, 79). The role of MDSCs in sepsis appears to be time-dependent in septic mice (87). For example, in one study, the adoptive transfer of day 3 MDSCs from septic mice into other septic mice resulted in an increased early mortality, whereas the adoptive transfer of day 12 MDSCs had a protective effect. In other studies, blocking MDSC expansion in septic mice resulted in an increased cytokine production and an increased mortality (87, 183).

After sepsis, human MDSC expansion may persist 28 days after the onset of sepsis (10, 11, 181, 184). Acute and chronic expansion of MDSCs after sepsis is associated with an increased incidence of nosocomial infection, adverse in-hospital outcomes, and poor discharge dispositions (10, 11). However, the mechanism(s) behind these adverse outcomes associated with an increased MDSC expansion are still poorly understood.

## CCI is the Predominant Phenotype of Innate Immune Activation in Trauma and Sepsis Survivors

In previous eras, a substantial proportion of patients who survived severe traumatic injury or septic insults were vulnerable to a non-acute death due to multiple organ failure. In modern ICUs, these patients often do not develop multiple organ failure and instead are subjected to the chronic smoldering organ failure of CCI. As previously discussed, DAMPs and PAMPs bind multiple PRRs, including TLRs, CLRs, NLRs, RLRs, and RAGE, with multiple redundant and converging pathways promoting a robust inflammatory response and emergency myelopoiesis, including the expansion and persistence of MDSCs (14, 185). In CCI, the persistent release of these endogenous alarmins perpetuates this inflammatory response and emergency myelopoiesis. Downstream of these pathways, septic patients who develop CCI have persistently elevated plasma sPD-L1, absolute lymphocyte counts, and monocytic HLA-DR expression compared with patients who exhibit rapid recovery (9). Chronic critical illness is associated with an increased incidence of nosocomial infections in both trauma and sepsis patients who develop CCI (9, 186). Efforts to better understand the pathophysiology of CCI among critically ill surgical patients are underway (187).

## PICS is the Final Common Denominator of Failed Resolution of the Innate Immune Response

Post-sepsis and severe injury patients, particularly CCI patients, often fail to achieve immune homeostasis. Rather than resolving the acute inflammation that accompanies these huge insults, these patients develop chronic low-grade inflammation. In addition to ongoing inflammation, these patients experience immune suppression and lean muscle wasting of prolonged duration. Collectively, this constellation of features has been coined PICS, or the persistent inflammation immunosuppression and catabolism syndrome (1, 8). Although this syndrome has only recently been defined, this phenomenon has been in existence for many years and is not limited to sepsis patients. Rather, PICS is thought to result from a wide array of pathological processes, including severe traumatic injury, extensive burns, and acute pancreatitis (1, 8, 188, 189).

A similar term that has been described is a series of conditions seen in survivors of ICU hospitalization, “post-intensive care syndrome” (190). “Post-intensive care syndrome” describes new or worsening impairments in physical, cognitive, or mental health status arising after critical illness and persisting beyond acute care hospitalization regardless of its etiology. Unlike the post-intensive care syndrome, the PICS has been proposed as *a mechanistic explanation* for the poor clinical outcomes observed in CCI patients by describing the immune dyscrasia that follows an inciting inflammatory response (1, 8, 190). PICS patients may well be the same “post-intensive care syndrome” patients, but the former describes an underlying hypothesis that is being tested experimentally, whereas the latter is a purely descriptive term. These PICS patients, irrespective of the term, are frequently discharged to LTACs and skilled nursing facilities where they often fail to rehabilitate, experience progressive declines in cognitive and functional status, require repetitive rehospitalization, and often result in indolent death (1, 8). Interestingly, a recent retrospective study, which investigated the clinical characteristics and long-term quality of life (QOL) after acute pancreatitis patients, determined that these patients can also enter the PICS. These patients had significantly lower scores in the QOL assessment questionnaire and a lower rate of returning to work as compared to those of “non-PICS” patients (189).

Recent work shows that patients who develop CCI after sepsis (i.e., patients with ICU LOSs of ≥14 days and ongoing organ dysfunction) exhibit persistent elevations in markers of inflammation out to 28 days after sepsis onset, including IL-6 and IL-8 (8). These patients also have elevated anti-inflammatory markers, such as IL-10, that persist out to 1 month. Along with elevations in pro-ILCs and anti-ILCs, CCI patients demonstrate reduced absolute lymphocyte counts, decreased HLA-DR expression on CD14^+^ monocytes, and elevated plasma concentrations of sPD-L1, all of which suggest impaired host immunity (9, 186). Clinically, this immune suppression manifests as an increased incidence of secondary infections and a 6-month mortality in the CCI group compared with rapid recovery patients. Finally, to support a decreased anabolic activity and an increased catabolism, CCI patients demonstrate decreased levels of albumin and insulin-like growth factor-binding protein 3, as well as elevated urinary 3-methylhistidine to creatinine ratios.

Although these cytokines and protein biomarkers serve as signatures for PICS, the detailed mechanisms leading to this persistent inflammation, immune suppression, and catabolism have yet to be elucidated. The discovery of the underlying mechanisms will undoubtedly require further studies, including additional genomic and proteomic analyses, as well as the creation of murine models of PICS, which are currently underway (191–193). Future success in the treatment of CCI will likely require addressing the underlying pathophysiology of PICS, potentially through the use of immunomodulatory therapies. Whether these attempted therapies will impact clinical outcomes is yet to be determined. Ongoing clinical trials testing therapies such as PD-L1 inhibitors in severe sepsis patients may provide further direction. Ideally, these therapies will directly impact dysregulated host immunity and prevent the perpetual cycle of PICS.

### Age Modulates Innate Immunity and the Host Response to Microbial Infections

The number of aged individuals around the world is dramatically rising (194, 195). Since the elderly population is expanding, research regarding this population has become increasingly relevant, especially with the escalating economic and health-care burdens in our society. Older patients are more likely to become critically ill and more likely to develop CCI. The causes of this phenomenon include senescence (normal aging), inflammaging (chronic subclinical systemic inflammation), comorbid conditions, lack of physiological reserve, and disability as well as environmental and epigenetic factors. These elements prevent older individuals from returning to a homeostatic state (194–197). In addition, one-third of all aged individuals suffer from frailty, a dynamic and comprehensive measure of physiologic age rather than simple chronological age (194, 195). All of these factors contribute to pathophysiology in the host’s organ systems, increasing the risk of disease, disability, and death in older patients, particularly within the first year after the onset of critical illness (194, 195).

Severe infection and severe injury illustrate the contribution of aging to poor outcomes. For example, sepsis is primarily a disease of the elderly. While the frequency of hospitalizations with sepsis for patients aged 18–49 years has barely changed from 2000 to 2007, the frequency of hospitalization with sepsis for patients aged 50–64 years has increased and has risen dramatically for patients aged 65 years or greater (198). Overall, in-hospital sepsis mortality is decreasing, but remains significantly higher in the elderly, and this cohort is more likely to be discharged to skilled nursing and rehabilitation facilities rather than home (1, 198, 199). A similar phenomenon is observed in the realm of geriatric trauma (200, 201). Although trauma is primarily a disease of the young, the volume of geriatric trauma is increasing (202). Advanced age in trauma is associated with a more severe organ failure, infectious complications, increased ventilator days, a longer ICU LOS, an increased 28-day mortality, and an increased likelihood of discharge to skilled nursing or long-term care facilities (203).

**Figure 3:**
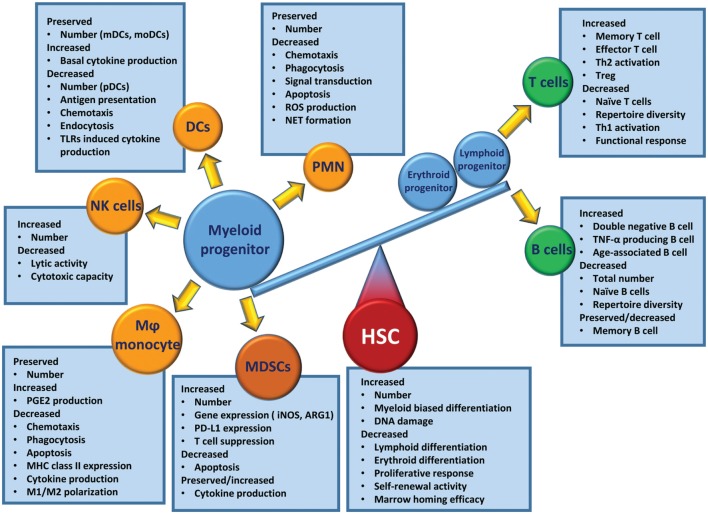
Immunosenescence in hematopoietic stem cells (HSCs), innate, and adaptive immune cells.

When regarding the poor outcomes of elderly patients after critical illness, one must consider the prevalence of chronic disease in the elderly and how modern medicine allows individuals with severe chronic diseases to survive into old age (1, 198, 204). In addition, age-related alterations in the immune system play a profound role in these poor outcomes. Immunosenescence is a state of age-associated changes in the immune system. Compared to the young, the aged immune system is less able to mount an effective response after challenges with infectious pathogens (205, 206). The competency of the adaptive immune system decreases with age, as evident by decreases in naïve peripheral T cells, repertoire diversity, and immunocompetent B cells (205, 207). Although aged host HSCs preferentially induce myelopoiesis, the effector cells engendered (i.e., PMNs, monocytes/Mφ, DCs, and NK cells) are dysfunctional compared with leukocytes from young individuals (206) (Figure 3).

Inflammaging, defined as a low-grade chronic systemic inflammation established during physiological aging, contributes to all the above conditions seen in the elderly (208). However, one should avoid the misconception that the term inflammaging describes how acute and subacute immune and inflammation responses of aged mammals are merely exacerbations of what is demonstrated in the young. Rather, the aged responses to sepsis and trauma are dissimilar to that of juveniles—a “hyper-cytokine storm” is either markedly attenuated or is not the direct cause of increased morbidity and mortality in the elderly after severe infection or injury (12, 13, 203). From progenitor to downstream effector cells, the aged response to severe infection or injury deviates from the response observed in younger counterparts.

In murine models, both trauma and sepsis induce a rapid release of mature and immature myeloid cell populations from the BM in response to endogenous and exogenous danger signals (87, 209). This evacuation of cells creates niches in the BM that stimulate emergency myelopoiesis, an endogenous effort to restore adequate numbers of myeloid populations (75). Myelopoiesis is driven at the expense of lymphopoiesis and erythropoiesis (75, 79). Interestingly, elderly HSCs have this phenotype and function prior to critical illness. Aged HSCs (specifically LT-HSCs) have myeloid-skewed cell production and a decreased ability to produce lymphoid cells (210, 211). The number of BM HSCs increases with age, and these HSCs are functionally inferior to their younger counterparts. They have a lower functional frequency, a delayed proliferative response, and a reduced efficiency for short-term BM homing, and they produce smaller clones of mature cells and have a reduced long-term self-renewal activity (210, 211). These HSC dysfunctions are further exacerbated by acute inflammatory insults, such as trauma and sepsis, which induce emergency myelopoiesis (12, 13). While the young are capable of returning to a balanced state of innate and adaptive immunity, elderly patients have difficulty returning to homeostasis (203). This results in the elderly having continued specific defects in their effector immune cells.

### Like Aging, Cancer Patients Are More Susceptible to PICS

Many analogies can be made among cancer, aging, and PICS patients. This includes the host innate immune system in each of these conditions. If one asks, “what induces a state of persistent inflammation, immunosuppression and catabolism, as well as being associated with frailty and poor outcomes?” the answer could be cancer, aging, or critical illness. Thus, it is not surprising that all three are related. Both the elderly and cancer patients are more likely to become septic and develop critical illness with poor outcomes (194, 195, 212). In addition, the elderly are more likely to develop cancer for the same reasons that they are less able to tolerate injury or infection (213, 214). Finally, any patient with CCI may develop a systemic phenotype that is similar to that of aged individual or cancer patient, which in its extreme form is classified as PICS (1, 8, 185).

**Figure 4:**
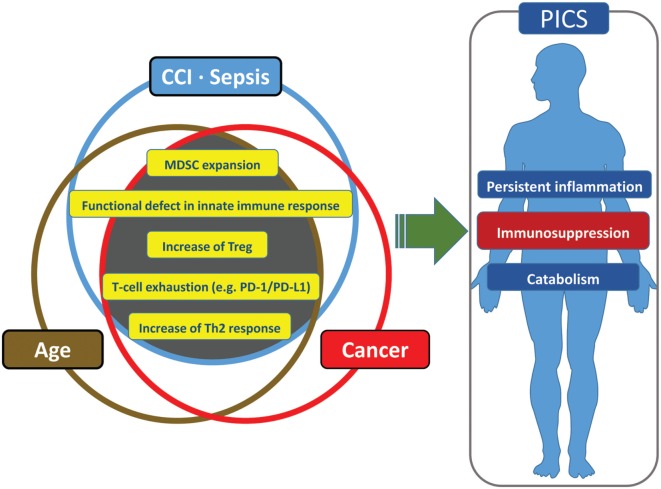
Similarities and redundancies in the pathophysiology of patients with sepsis, cancer, and advanced age.

It is not surprising that cancer patients have an immune environment similar to that of the aged and the CCI population and that this immune status is thought to engender poor outcomes. Cancer patients have persistent antigen exposure and protracted inflammation which eventually lead to pathological effects on host-protective immunity as well as tissue wasting and cellular apoptosis (185). Cancer cells can recruit immune cells to their local environment through the release of local and systemic immunosuppressive mediators (185). Neoplasia can induce immunosuppression through multiple other mechanisms and effector cells, including myeloid-derived suppressor cells, M2 macrophages, T-cell exhaustion, T regulatory cells, and expression of inhibitory ligands (185). Some have gone so far as to say cancer cells “hijack [host immune] tolerance [and suppression] (215).” It is for these reasons that many researchers are attempting to use approved cancer immunomodulation agents for sepsis therapy (e.g., anti-PD-1/PD-L1 Ab, anti-CTLA-4 Ab) (185) (Figure 4).

Regulation of the hematopoietic response to stress is an integral part of innate immunity (215). Most HSCs are somewhat quiescent, participating in the maintenance of homeostasis (216). However, after injury to the host (e.g., cancer, infection, trauma, burn), HSCs become active, reenter the cell cycle, and undergo differentiation (215). This activation can occur indirectly through mesenchymal or immune cells, or directly through exogenous or endogenous ligands (217, 218). Chronic exposure to factors that induce stem cell activity induces HSC defects, including their ability to repopulate (219). One result of this HSC environment is the creation of MDSCs (215). MDSCs represent a wide range of cells in various stages of differentiation and are generated in particular when myelopoiesis is amplified over lymphopoiesis and erythropoiesis (215). The role of MDSCs is still unclear, as their immunosuppressive properties are vital for the resolution of acute inflammation, but they also may play a pivotal role in emergency myelopoiesis in conditions like injury or infection (87, 215). Regardless of this paradox, their chronic persistence is associated with poor outcomes in both cancer and sepsis patients (8, 10, 215).

## Future for Precision Medicine and Therapeutics

### Immune-Modulatory Therapies in Sepsis

Sepsis is associated with alterations in immune effector cells including defects in antigen presentation, quantitative and qualitative alternation in neutrophils, defective NK cell-mediated immunity, defective T and B cell-mediated immunity, relative increases in regulatory T cells (Tregs), an increased expression of PD-1/PD-L1, decreased immunoglobulin levels, hypercytokinemia, and complement consumption. Immune-modulatory therapies for sepsis are now being investigated, with a focus on the restoration of immune system homeostasis. Agents under consideration include leukocyte growth factors (e.g., granulocyte macrophage colony-stimulating factor and G-CSF) (220–224), immunomodulatory cytokines (e.g., IL-7, IL-15, and IFN-γ) (225–229), inhibitors of negative co-stimulatory pathways (e.g., anti-PD-1/PD-L1 Ab, anti-CTLA-4 Ab, anti-TIM3 Ab, and anti-LAG-3 Ab) (230–236), and the thymic peptide thymosin-α1 (237). Although some of these agents have previously failed as “silver bullet” curative monotherapies, many have been associated with partial improvements. For example, two randomized clinical trials involving recombinant G-CSF, which is intended to increase neutrophil production and microbial eradication, demonstrated an increase in total leukocyte counts in patients receiving the experimental therapy (220, 221). Since emerging evidence suggests that the phenotype of CCI/PICS patients is somewhat similar to that of cancer patients, it is possible that emerging oncologic therapeutic targets and strategies could be successful in treating CCI/PICS.

### Nutritional Support and Exercise

The loss of lean body mass is one of the clinical characteristics of CCI patients. A prolonged low-grade inflammation in muscle may lead to catabolism and myonecrosis that result in the loss of lean body mass (238, 239). Thus, nutritional support and exercise are pivotal components in the care of CCI patients. Among CCI patients, optimal amounts of protein intake may be more important than the total daily caloric intake in promoting recovery. However, optimal daily protein administration thresholds for CCI patients have not been elucidated. If CCI patients have similar metabolic requirements as patients with cancer cachexia and aging sarcopenia, then the recommended daily protein intake would be 1.5–2.0 g/kg/day (240–244).

Candidate therapies for nutritional therapy in CCI include arginine, leucine, and anabolic adjuncts. The use of arginine in sepsis is controversial because arginine serves as an intracellular substrate for nitric oxide, which may cause pathologic vasodilation. However, the upregulation of arginase-1 in MDSCs has been observed following trauma and sepsis (10, 180), which may result in loss of arginine. Arginine is necessary for T-cell receptor function, as well as ornithine, which is converted to polyamines and prolines that are necessary for wound healing and tissue repair (245–247). Therefore, with MDSC expansion and persistence during PICS, arginine may have the capacity to restore lymphocyte proliferation and promote wound healing and tissue repair. Leucine is one of several branched-chain amino acids (BCAAs), which decreases muscle protein catabolism and induces protein synthesis (248). In a prospective randomized trial, the use of BCAA-fortified parenteral nutrition in surgical patients improved several nutritional and immunological parameters such as nitrogen balance, prealbumin levels, and lymphocyte counts (249). Moreover, recent studies demonstrated that leucine supplementation improves muscle protein synthesis in the elderly and in cancer patients by stimulating the mammalian target of rapamycin (mTOR) pathway (250, 251). Thus, leucine or BCAA supplementation may be beneficial in PICS. In addition, several adjuncts such as insulin, oxandrolone, and propranolol have been shown to maintain the anabolic state in patients with burns. These therapies may also be effective in mitigating the catabolic component of PICS (252–254).

Finally, there has been an increasing interest in the functional status and health-related QOL in CCI patients. Early ICU-based exercise and rehabilitation programs have been associated with a reduction in the duration of mechanical ventilation and ICU LOS, as well as improved physical function (255). Studies in non-ICU populations have demonstrated that the combination of exercise and nutritional support has the largest treatment effect on protein synthesis and physical strength compared with nutrition or exercise alone (256). Although the effect of this combination therapy in CCI patients is unknown, the pathophysiology of CCI and PICS suggests that similar exercise and nutritional programs may be useful in these populations as well.

### Microbiome

Since the 1980s, it has been hypothesized that dysregulated crosstalk among the epithelium, immune system, and gut microbiota leads to the development of sepsis and multiple organ failure. In the last decade, a number of studies have produced valuable insights into the pathophysiologic mechanisms responsible for these phenomena (257). Interactions among intestinal epithelial cells and microbiota through signaling of PRRs such as TLRs and NLRs and microbiota-derived short-chain fatty acids (SCFAs) appear to influence both local and systemic immunity. The microbiota influences the development, maturation, and function of myeloid cells in multiple organs and tissues including lungs, intestines, BM, and circulating myeloid cells. For example, germ-free mice demonstrate the reduced development of myeloid cell progenitors in BM (258), and continuous TLR stimulation by commensal microbiota drives neutrophil development (259).

In sepsis, several changes occur in gut physiology and immunity, including loss of gut motility, increased bowel wall permeability, and apoptosis of intestinal epithelium due to extrinsic factors (e.g., antibiotics, opiates, and parental and enteral nutrition) and intrinsic factors (e.g., inflammation and increased bowel wall permeability). These changes may result in the alternation of the microbiota composition (260, 261), overgrowth of pathogenic microbes (262), and loss of commensal organisms (263). In patients with the systemic inflammatory response syndrome, there is a loss of diversity in gut microbiota, which is associated with an increased incidence of bacteremia and an increased mortality (260).

However, high-level evidence and recommendations regarding therapeutic modulation of gut microbiota for septic patients are lacking. Several candidate therapies are under investigation, including pro/pre/synbiotics (263), l-glutamine (264), fecal microbiota transplantation (265), and phosphate (Pi) (266). Currently, there is limited evidence to support the use of these therapies for patients with sepsis, and further investigation is warranted.

### The Use of Big Data

The use of big data in medicine has been rapidly evolving. This approach has the potential to provide researchers and clinicians with the information necessary to practice precision medicine tailored to individual patients. For this purpose, there are many databases such as The Human Genome Project (available from: www.genome.gov), Encyclopedia of DNA Elements (a project to identify all functional elements in the human genome sequence; available from: www.encodeproject.org), Roadmap Epigenetics Project (available from: www.roadmapepigenomics.org), as well as the “Inflammation and Host Response to Injury” Glue Grant (available from: www.gluegrant.org). Big data has already yielded important advances in the field of medicine. For example, tumor transcriptomics are being used to predict pharmacologic and therapeutic responsiveness for some forms of leukemia and solid tumors.

Big data is particularly useful in the translational research approach to sepsis due to the heterogeneity of sepsis populations, the limited utility of individual biomarkers, and the wide variability in the duration and severity of illness prior to presentation. For the same reasons, genomic, metabolomic, and transcriptomic markers may be of great value in generating prognostic models and identifying optimal candidates for tailored therapies (267–269). Recently, it has been reported that the leukocyte transcriptome in trauma patients may predict outcomes using samples obtained within 48 h of injury (270). This study and similar studies using regression-based prediction models may be further improved by the use of machine-learning algorithms and deep-learning technologies (271, 272). Although big data provides sample sizes large enough to identify biomarker cutoff values with optimal sensitivity and specificity for the outcome of interest, these static variable thresholds based on aggregate patient populations fail to account for individual patient physiology. Therefore, future efforts should continue to evolve precision medicine approaches for septic patients by integrating data from multicenter and multinational repositories with machine-learning and deep-learning technologies that recognize and manage complex, chaotic, and nonlinear associations among input variables.

## Conclusion

Following remarkable advances in our understanding of the pathophysiology and natural history of trauma and sepsis, two major targets for improving outcomes remain: early death and indolent death attributable to CCI and PICS. A better understanding of the pathophysiologic mechanisms responsible for CCI and PICS may allow for the identification of novel management strategies and therapeutic targets. Future research should continue to investigate anti-inflammatory agents, immune modulators, gut microbiota support, as well as nutritional and exercise therapy. These should all be guided by validated prognostic models, hopefully taking advantage of novel “big data” datasets, to identify patients for whom these approaches will yield the greatest benefits. Retrospective “community” big data can also be used to identify appropriate biomarkers, surrogate outcomes, and putative research approaches. Randomized clinical trials, using biomarker-driven adaptive study design, are also more rapidly moving potential therapeutics into the clinical setting.

## Author Contributions

HH, TL, RH, SR, JS, and PE drafted the manuscript. MH, BW, EM, AB, SL, AM, SB, HT, HU, FM, LM, and PE provided critical revisions. All authors made substantial contributions to the conception and design of the work, approved the submitted version of the manuscript, and agree to be accountable for all aspects of the work.

## Conflict of Interest Statement

The authors declare that the research was conducted in the absence of any commercial or financial relationships that could be construed as a potential conflict of interest.
